# Treatment of Nongout Joint Deposition Diseases: An Update

**DOI:** 10.1155/2014/375202

**Published:** 2014-05-08

**Authors:** Tristan Pascart, Pascal Richette, René-Marc Flipo

**Affiliations:** ^1^Department of Rheumatology, Saint-Philibert Hospital, Service de Rhumatologie, Lille Catholic University, rue du Grand But, 59160 Lomme, France; ^2^Department of Rheumatology, Roger Salengro Hospital, Lille 2 University, rue Emile Laine, 59037 Lille Cedex, France; ^3^Federation of Rheumatology, Lariboisiere Hospital, Assistance Publique-Hopitaux de Paris, UFR Medicale, Paris 7 University, 75475 Paris Cedex 10, France

## Abstract

This update develops the actual therapeutic options in the management of the joint involvement of calcium pyrophosphate deposition disease (CPPD), basic calcium phosphate (BCP) deposition disease, hemochromatosis (HH), ochronosis, oxalosis, and Wilson's disease. Conventional pharmaceutical treatment provides benefits for most diseases. Anti-interleukine-1 (IL-1) treatment could provide similar results in CPPD than in gout flares. There is only limited evidence about the efficacy of preventive long-term colchicine intake, methotrexate, and hydroxychloroquine in chronic CPPD. Needle aspiration and lavage have satisfactory short and midterm results in BCP. Extracorporeal shockwave therapy has also proved its efficacy for high-doses regimes. Phlebotomy does not seem to have shown real efficacy on joint involvement in HH so far. Iron chelators' effects have not been assessed on joint involvement either, while IL-1 blockade may prove useful. NSAIDs have limited efficacy on joint involvement of oxalosis, while colchicine and steroids have not been assessed either. The use of nitisinone for ochronotic arthropathy is still much debated, but it could provide beneficial effects on joint involvement. The effects of copper chelators have not been assessed either in the joint involvement of Wilson's disease. NSAIDs should be avoided because of the liver affection they may worsen.

## 1. Introduction

New interest in crystal-induced arthropathies has developed over the last few years mainly through the new discoveries on gout pathophysiology and especially with the understanding of the inflammasome [1] which paved the way for new therapeutic options [2]. These breakthroughs in the field of gout have given a new insight into other crystal-induced arthropathies [3] and recent international guidelines have been produced for both the treatment of gout [4, 5] and calcium pyrophosphate deposition (CPPD) [6]. However, other crystal-induced rheumatisms such as the basic calcium phosphate (BCP) deposition disease [7] do not seem to have benefited from these new developments although they are widespread in clinical practice.

Rare conditions such as hemochromatosic arthropathy [8], ochronotic arthropathy [9], Wilson's disease [10], and oxalate crystal deposition disease [11] can lead to difficult clinical situations. Other joint deposition-related entities such as cholesterol crystal arthropathy, cryoglobulin-crystal arthropathy, liquid lipid crystals arthropathy, corticosteroid crystals-induced arthritis, or Charcot-Leyden crystals deposition are exceptional and therefore not developed here. Crystal-induced arthropathies, and more generally deposition diseases, share similarities in their treatment but also have distinctive features. To our knowledge, no recent global overview of the treatment of deposition-induced rheumatisms has been published in international literature [12, 13]. The main objective of this literature review is to provide a general update of the actual available treatments for deposition-induced arthropathies.

## 2. Methods

MEDLINE (medical literature analysis and retrieval system onLine) was used to pick out relevant articles up to January 2014. The keywords “treatment,” “management,” “guidelines,” “recommendations,” “colchicine,” “nonsteroidal anti-inflammatory drugs,” “Anakinra,” and “corticosteroids” were successively associated with the terms “deposition,” “crystal-induced,” “micro-crystalline,” “deposition,” “arthropathies,” or “arthritis” and with the name of the different deposition disease (e.g., for “calcium pyrophosphate deposition,” the key words “chondrocalcinosis,” “calcium pyrophosphate dehydrate crystals,” and “pseudogout” were also used). Initial article selection was based upon titles and abstracts. Cross-references from the found articles were also explored. Articles written out in languages other than English were studied for information when relevant but their data were not included in the review.

## 3. Results and Discussion

**Figure 1:**
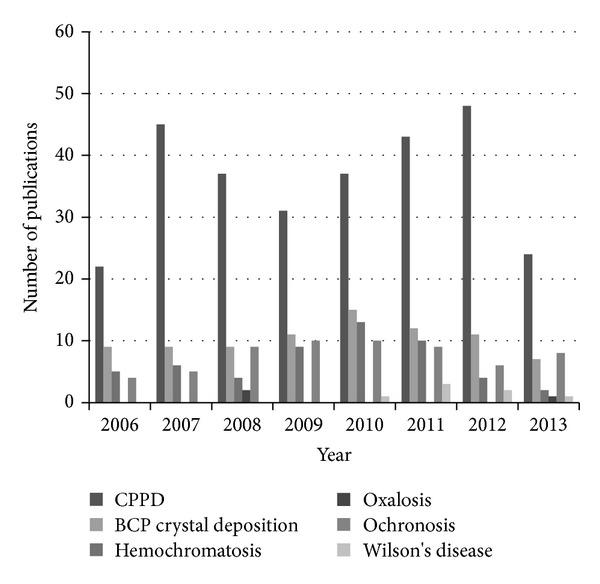
Number of publications per year related to management or treatment of each joint deposition disease from 2006 to 2013. CPPD: calcium pyrophosphate deposition; BCP: basic calcium phosphate.

CPPD shows the highest number of publications amongst other deposition diseases which are the subject of around thirty to forty articles per year. Incidentally over the same time period, an average of 258 publications per year dealt with the issue of the management of gout (Figure 1).

### 3.1. Calcium Pyrophosphate Deposition (CPPD)

CPPD is widespread and its prevalence increases with age [14]. It is therefore fundamental to take cardiovascular and renal comorbidities into account, frequent amongst the elderly, when pharmacological treatment is considered. Relatively close to gout in terms of mechanisms and treatment habits, some new therapeutic options inspired by the discoveries in gout are emerging in the CPPD.

#### 3.1.1. Treatment of CPPD Flares

The EULAR recently defined new guidelines for the management of CPPD [6]. Radiological findings of chondrocalcinosis do not motivate any particular management. Treatment should be limited to symptomatic CPPD. Local measures with ice bladders can provide some pain relief. Data is more limited regarding treatment of CPPD flares than of gout, but it is agreed that treatment of the crisis relies on the use of colchicine or NSAIDs. Considering the frequent side effects of colchicine and the renal impairment observed in this elderly population, no loading dose is recommended. Intra-articular injection of corticosteroids is an efficient and well tolerated therapeutic alternative. Oral or parenteral corticosteroids can be used in case of refractory flares. Retrospective data suggests satisfying efficacy of ACTH [15] with a rapid control of the symptoms after a 1 mg intramuscular injection of 13 out of 14 patients without any particular adverse effect. However, no information is given regarding the length of the follow-up. Evidence of the efficiency of anti-IL1 treatments (Anakinra) is growing but data is still limited and this option is not yet recommended, though it seems a good alternative in case of poor response or counterindications of NSAIDs, colchicine, and steroids, especially in case of polyarticular flare. The largest series included 16 cases among which two-thirds of the patients showed a satisfactory response [16]. Twelve patients were treated according to a protocol of daily subcutaneous administration of 100 mg Anakinra, the remaining four patients being treated from one week up to six months. Relapse occurred after treatment discontinuation in around one patient out of three, such results are similar to those observed in a case series of 40 gouty patients in which 13 cases experienced relapse [17]. Incidentally, 11 patients received simultaneous oral corticosteroids with various levels of prednisone and various prophylactic treatments were given to six patients after the flare, making it more difficult to assess the ability of Anakinra to cure the flare and prevent relapse.

#### 3.1.2. Treatment for Chronic CPP Crystal Inflammatory Arthritis

Daily 1 mg intake of colchicine is sometimes used but there is too restricted data to prove its efficiency [18]. In fact, we can rely on single dated study including ten patients which showed a three-fold decrease of flares over a year of follow-up after they had received oral colchicine in comparison with the previous year without treatment [19]. Further evidence on a subset of patients affected with CPPD and knee osteoarthritis showed a symptomatic benefit after five months of a 1 mg colchicine daily intake added to initial treatment by piroxicam and intra-articular steroid injection compared to placebo [20]. However, only 74% of the patients included in the study had demonstrated CPPD crystals with synovial fluid examination. Although seldom used in clinical practice, the EULAR underlined the possible benefits of methotrexate [21] and hydroxychloroquine [22] in prophylaxis of CPPD flares. However, the study of 5 patients with an average follow-up of 6.2 years [21] upon which EULAR's recommendations for the use of methotrexate are based was contradicted by a report of three nonresponsive patients at 6 and 12 months [23]. A more recent observational study including ten patients suggested possible benefits of methotrexate; however, primary endpoints of the study were limited to a subjective assessment by the physician or the patient himself [24]. No further randomized controlled study has been performed so far. Guidelines regarding the use of hydroxychloroquine rely on a 6-month double-blind placebo-controlled study including 36 patients and showing some rapid benefits on the number of swollen and tender joints [22]. Magnesium intake in patients with hypomagnesaemia could reduce the number of flares and slow down radiographic progression of CPPD [25] as suggested in a dated placebo-controlled study including 38 patients [26]. Case reports suggest an inefficiency of Anakinra on chronic manifestations of CPPD [27] especially in the absence of systemic inflammation [28]. CPPD treatment is essentially medical but surgical treatment has been carried out successfully for the management of temporomandibular involvement [29, 30].

### 3.2. Basic Calcium Phosphate (BCP) Crystal Deposition Disease

Tendinitis and bursitis, especially of the shoulder, due to BCP crystal deposits are very frequent affections [7]. BCP deposits can also take place in intra-articular localizations associated with highly destructive arthritis, amongst which the most described form is the Milwaukee shoulder [31].

#### 3.2.1. Treatment of Periarticular BCP Crystal Deposition Disease

**Figure 2:**
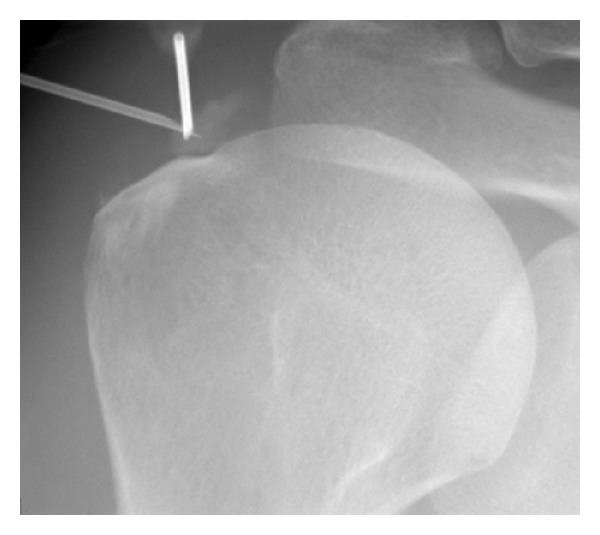
Radiologically guided aspiration of a calcification of the supraspinatus.

In case of acute tendino-bursitis, symptomatic treatment with ice, joint rest, systemic NSAIDs, and periarticular corticosteroid injection is usually proposed [32]. Systemic corticosteroids at a dose of 30 mg per day of prednisone could be useful but this classical recommendation relies essentially on experts' clinical experience. In case of refractory tendinitis with large calcifications, ultrasound guided needle aspiration and lavage of the BCP deposit, followed by periarticular corticosteroid injection, show interesting results after one month, three months, and one year in both large nonrandomized controlled trials including 289 patients [33] and smaller randomized controlled studies [34]. This strong level of evidence suggests that needle aspiration and lavage should be proposed to patients with large calcifications refractory to conventional treatments. As expected, given the natural history of basic calcium deposits, no difference was found, however, on the long term (five and ten years), both groups of patients having benefited from a similar favorable outcome (Figure 2). The procedure can induce a painful flare due to the resorption of the calcification, which can justify a prophylaxis by colchicine or NSAIDs. Extracorporeal shockwave therapy could be used in case of failure of the preceding treatments as suggested by a randomized placebo-controlled trial conducted among 144 patients [35]. Reviews and meta-analyses suggest that proof of effectiveness of such treatment is limited to the shoulder and essentially with high-doses regime patients [36, 37]. Additional prospective studies using high-doses regime seem necessary to firmly recommend a more systematic use of shockwave therapy. Ultrasound therapy showed some possible benefits but too limited data is available apart from a prospective study of 63 patients which showed satisfactory results on the short term [38]. However, the sequence of administration of the ultrasound therapy was intense (daily administering) which is difficult to sustain in common practice. Yet, given the good tolerance profile of ultrasound therapy, although its efficacy when used only several times weekly, acceptable frequency in common practice, has not been thoroughly explored, this option seems interesting in a combined approach. Isolated data reports a possible benefit from IL-1 blockade. Indeed, an open-label study of five patients showed rapid control of the symptoms with relapse at the end of the treatment for a single patient [39]. Refractory calcifications to medical treatment responsible for recurrent flares can lead to surgical removal usually by arthroscopy [32, 40], but this option should be limited to situations where exhaustive medical treatment was ineffective.

#### 3.2.2. Treatment of Intra-Articular BCP Crystal Deposition Disease

Symptomatic treatment by analgesics and NSAIDs is usually proposed with the injection of intra-articular steroids. In the long term, treatment is similar to any destructive arthritis with physical preservation of the joint and eventually joint replacement. Tidal lavage of joints has been studied on a small group of ten patients with better results on patients with recent onset of Milwaukee shoulders [41]. However, the natural evolution of the disease remains unclear and the efficacy of such intervention is argued. A recent case report suggests possible efficacy of oral corticosteroids [42] (Table 1).

### 3.3. Hereditary Hemochromatosis (HH)

**Figure 3:**
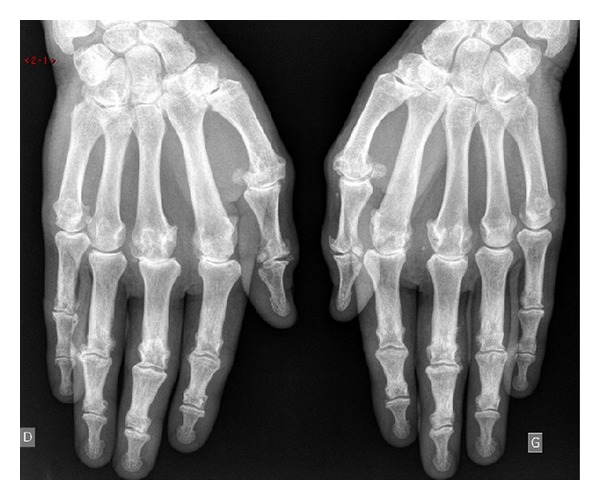
Radiological manifestations of the hands of hereditary hemochromatosis.

HH is a cause of secondary CPPD but is also responsible for osteoarticular manifestations by itself which can reveal the disease [43]. Joint involvement is the first cause of the degradation of the quality of life of patients suffering from HH [44] (Figure 3).

Limited data regarding treatment of the joint involvement of HH exist. Treatment relies essentially on symptomatic measures with the use of analgesics and NSAIDs. Colchicine can be useful during flares most probably due to CPPD-associated involvement. Intra-articular injections of corticosteroids can be used but no relevant published data exist on the issue. Some data suggest the possible efficiency of phlebotomy but its effects, if any, this is still highly debated, are unpredictable [45]. Indeed, in an observational study of 199 patients of whom 132 underwent phlebotomy, 13.6% of the patients reported improvement of joint pain, 65.9% reported no change, and 20.5% experienced a worsening of their articular symptoms after phlebotomy. This is further supported by the fact that iron depletion has itself a paradoxical increasing effect on collagen II levels indicative of cartilage degradation [46]. Thus, given the actual contradictory available data, phlebotomy should not be proposed for articular purposes. Iron chelators, deferasirox in particular, have shown some efficiency when phlebotomy is contraindicated [47] but effects on joint involvement have not been assessed. Furthermore, some recently reported serious adverse effects question the overall safety of the drug [48]. Interleukin-1 receptor antagonist has been shown to be effective in some patients with refractory hemochromatosis-related arthritis of the hands [49] but these data remain very limited. Natural evolution can lead to joint replacement [50].

### 3.4. Oxalate Crystal Deposition Disease

Skeletal involvement of oxalosis is found almost exclusively in evolved primary hyperoxaluria [12] (genetic overproduction of oxalate) as opposed to secondary oxalosis (essentially due to chronic kidney disease or increased intestinal absorption). Oxalosis is responsible for bone pain and pathological fractures whereas joint involvement shows acute arthritis (especially of the hands) similar to other crystal-induced joint flares [11, 51, 52] but also tenosynovitis of the feet.

#### 3.4.1. Treatment

NSAIDs seem to have very limited efficacy on oxalate arthritis in case reports [52, 53]. No evidence on the effect of colchicine or steroids (intra-articular or systemic) has been reported. General treatment of the disease can bring some benefits on the joint involvement. Diet modifications have no impact on the course of primary hyperoxaluria. Pyridoxine intake can partially correct the enzymatic defect, but eventually treatment leads to liver-kidney transplantation, which can sometimes be delayed by higher hydric intake, alkaline citrate, magnesium, and orthophosphates [54]. Recent data have suggested a possible efficacy of Anakinra on oxalate nephropathy [55] in mice models but have not yet been assessed in joint involvement. Liver cell transplantation, gene therapy, and use of chemical chaperones are leads towards new therapeutic options [54].

### 3.5. Ochronotic Arthropathy

**Figure 4:**
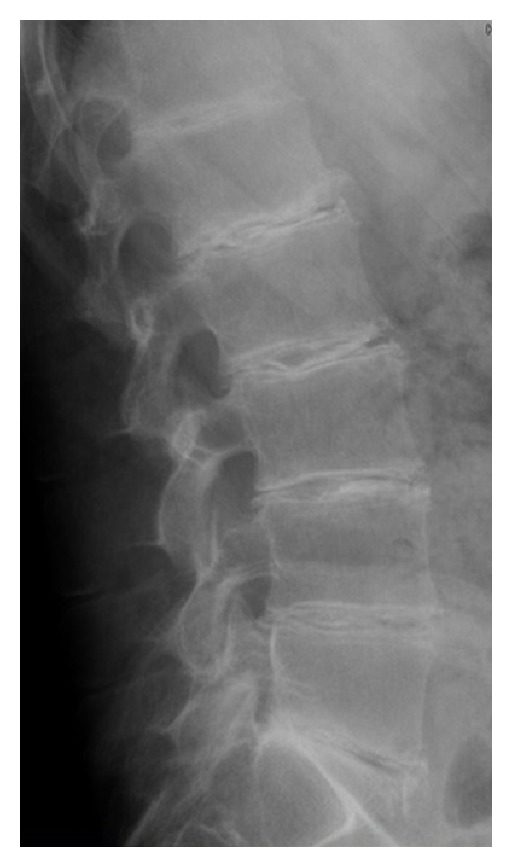
Ochronotic spinal deposits.

Ochronosis is due to a congenital defect in homogentisic acid oxidase leading to homogentisic acid accumulation and its deposition, especially inside joints [9], leading to chondrocyte death and matrix degradation. Spondyloarthritis-like spinal involvement usually precedes peripheral ochronotic arthropathy [56] (Figure 4).

#### 3.5.1. Treatment

Pain control is essential and difficult. It relies on both pharmaceutical (conventional analgesics, NSAIDs, and anticonvulsants) and physical measures [9, 57, 58]. Intra-articular injections of corticosteroids and hyaluronic acid have been used in case reports with varying immediate results but no sustained efficacy [59]. Joint replacement is sometimes needed. Spinal surgery for complications especially spinal stenosis must be carried out according to case reports [60].

No treatment to compensate the enzymatic defect has been developed so far. Diets excluding tyrosine and phenylalanine have no effect on developed ochronotic arthropathy. However, restricted protein intake associated with ascorbic acid ingestion could show some benefits. Treatment by nitisinone has shown some results in biological outcomes [61]. Yet, it remains debated especially since the only available randomized controlled trial including 40 patients, though well-tolerated, could not show any efficacy on the primary and secondary clinical outcomes [62]. However, some patients experienced clear improvement in joint symptoms and some authors suggest the need for further trials on nitisinone [63]. The study also suggested the importance of physical medicine. A recent animal model of alkaptonuria brought further evidence in favor of nitisinone for the prevention of the development of ochronotic arthropathy [64]. Liver [65] and renal [66] transplantation for other causes have shown beneficial effect on alkaptonuria according to case reports but with few or no details on the evolution of the joint involvement. New development of animal models could provide an interesting gateway for innovative therapeutic agents [67]. Recent advances in the understanding of ochronosis pathophysiology opened new therapeutic perspectives and suggest possible benefits of antioxidants [68].

### 3.6. Wilson's Disease

Wilson's disease (hepatolenticular degeneration) is responsible for visceral copper deposition. Articular manifestations are usually mild and affect large joints (especially knees) [10]. Precocious onset of osteoarthritis is also described.

There is no specific treatment of the joint involvement of Wilson's disease, apart from the copper chelators (D-penicillamine, zinc, and trientine) used to treat the general disease. Reviews showed a better efficacy for D-penicillamine though rheumatologic symptoms were not assessed and tolerance was poor [69]. Furthermore, D-penicillamine can be responsible for rheumatologic disorders by itself especially with cases of induced lupus [70]. The choice of treatment differs according to the disease's clinical presentation, mostly according to which hepatic or neurological symptoms prevail [71]. Diets restricted in copper seem unnecessary to the exception of liver and shellfish. Physical therapy is both beneficial on joint involvement and mostly on the neurological manifestations [72]. NSAIDs should be avoided due to the underlying liver affection; no data was found for colchicine and corticosteroids. There is one case report suggesting improvement of joint pain after liver transplantation [73] (Table 2).

## 4. Conclusion

The global interest in gout has provided advances in the management of the other joint deposition diseases, especially regarding Il-1 blocking treatments that seem efficient in CPPD, BCP, and HH. However, rather surprisingly, given the high prevalence of these diseases (especially CPPD and BCP), most of the data discussed in this study about their treatment rely on case reports and case series with isolated controlled trials. Further randomized controlled trials would bring considerable progress in the therapeutic management of joint deposition diseases.

## Figures and Tables

**Table 1:** Therapeutic options for CPPD and BCP deposition diseases compared to those available in gout.

	Gout	CPPD	BCP
Guidelines	ACR (2012) EULAR (2006) BSR (2007)	EULAR (2011)	None
Local treatment of the flare	Intra-articular corticosteroid injection	Intra-articular corticosteroid injection	Periarticular corticosteroid injection-calcification aspiration-shockwave therapy
Efficacy of colchicine in flares	Yes	Yes	Limited data
Loading dose of colchicine	Yes	No	—
Efficacy of NSAIDs in flares	Yes	Yes	Yes
Efficacy of systemic corticosteroids in flares	Yes	Yes	Limited data
First-line preventive treatments	Xanthine oxydase inhibitors	None	None
Second-line preventive treatment	Uricosurics	Little data on colchicine	—
Third-line preventive treatment	Recombinant uricase	Little data on methotrexate and hydroxychloroquine	—
Efficacy of anti-interleukine-1 treatments	Established	Possible	Possible

CPPD: calcium pyrophosphate deposition; BCP: basic calcium phosphate; ACR: american college of rheumatology; EULAR: European league against rheumatic diseases; BSR: British society for rheumatology.

**Table 2:** Treatment of rare deposits-induced arthropathies.

	Specific treatment of the joint involvement	Efficacy of colchicine	Efficacy of NSAIDs	Efficacy of systemic corticosteroids	Efficacy of intra-articular corticosteroid injection	Efficacy of interleukin-1 blocking agents	Other measures improving joint condition
Hereditary hemochromatosis	No	No data	Yes (limited data)	No data	No data	Yes	Phlebotomy (debated)
Oxalosis	No	No data	Poor	No data	No data	Possible	Pyridoxine—liver-kidney transplantation (no clear efficacy on arthropathy)
Ochronosis	No	No data	Yes (limited data)	No data	No data	No data	Limited protein intake and ascorbic acid—nitisinone (debated)
Wilson's disease	No	No data	No data	No data	No data	No data	Copper chelators (no clear efficacy on joint involvement)—liver transplantation (one case report)
